# Unclassified acetabular fractures: Do they really exist?

**DOI:** 10.1007/s00590-024-03908-8

**Published:** 2024-04-12

**Authors:** Mohammad Kamal Abdelnasser, Bahaaeldin Ibrahim, Mostafa A. Thabet, Ali Fergani, Mahmoud Badran, Osama Farouk

**Affiliations:** 1 Orthopedic Department, Assiut University Hospital, Assiut, Egypt; 2 Orthopedic Department, Al-Azhar University, Assiut, Egypt

**Keywords:** Hip, Acetabular fractures, Classification

## Abstract

**Purpose:**

Although Letournel classification is considered the corner stone for classifying acetabular fractures, however, it might not be perfectly inclusive. Unclassified fractures were reported by many authors. The aim of this case series is to report the incidence of unclassified acetabular fractures and description of these rare patterns and why they are considered unclassified acetabular fractures.

**Methods:**

This is a retrospective consecutive case series. In the period between 1st January 2016 and 31st December 2017, 235 patients with 236 acetabular fractures were identified from our hospital records. Classification of the acetabular fractures according to Letournel was done by two surgeons. Any discrepancy in the classification between the two surgeons was resolved by the senior author. Before considering the fracture unclassifiable, all fractures were reviewed again by the two surgeon and the senior author.

**Results:**

In the period between 1st January 2016 and 31st December 2017, 235 patients with 236 acetabular fractures were included in our study. Twenty-two fractures (9.3%) did not fit into any of the fracture types according to Letournel Classification as follows: 1 case (4.5%) was pure Quadrilateral plate fracture, 1 case (4.5%) was labral avulsion with tiny posterior wall rim, 1 case (4.5%) was pure articular impaction, 1 case (4.5%) was both columns fracture with posterior wall, 4 cases (18.2%) were anterior column and quadrilateral plate fracture, and 14 cases (63.8%) were T with posterior wall.

**Conclusion:**

Several acetabular fracture pattern could be considered unclassified fractures. These unique patterns may require special approaches or special fixation methods. However, this is not a call for a new classification for acetabular classification to include these new types. Subclassification or adding modifiers to Letournel classification can do the job.

## Introduction

Acetabular fractures are both complex in terms of classification and management. Historically, various classifications have been developed to guide surgical plan and fixation method. In 1951, Cauchoix and Truchet [1] categorized acetabular fractures into two main categories: central or posterior dislocation of the hip with fracture of the acetabulum. Creyssel and Schnepp in 1961 [2] further delineated acetabular fractures using principal and accessory fractures lines, while Rowe and Lowell in 1961 [3] utilized the radiologic acetabular dome as the main element for classification.

Most notably, letournel classification system, released in 1964 [4] and modified in 1980 [5], continues to be utilized to identify both elementary and associated fracture patterns with good inter- and interobserver reliability [6–8]. Letournel described acetabular fractures to be a continuous variable with the ten main types in their classification to be the most common type for each group of similar patterns of acetabular fracture. Under each category, they described a number of subtypes and atypical types. Letournel described 10 main types (5 elementary and 5 associated) and 52 subgroups that covers the transitional forms between different types.

Although acetabular fractures have historically been classified with 2D radiographs, however, the advent of advanced imaging techniques and combined injury mechanisms have created potential for recognition of acetabular fracture patterns that do not fit perfectly into the current standard classification schemes available [9–16]. Several epidemiological studies and case series reported the incidence of unclassified fractures between 1% up and 35% of all acetabular fractures admitted to their institutions [17–19].

The aim of this case series is to report the incidence and describe a number of unclassified patterns of acetabular fractures among patients admitted with acetabular fracture in two successive years in our institution and why they were considered unclassified acetabular fractures.

## Methods

This is a retrospective consecutive case series conducted at level-I trauma center. In the period between 1st January 2016 and 31st December 2017, 235 patients with 236 acetabular fractures were identified from our hospital records. After approval of our institutional review board, medical records and radiographic studies were reviewed. Routine imaging of acetabular fractures at our institution includes anteroposterior (AP) pelvic radiograph, oblique (Judet) views, and computed tomography scanning (CT) including 3D reconstruction. CT allows precise delineation of the fracture lines and assessment of articular impaction injuries and intraarticular fragments. Data collected included age, sex, mechanism of injury, co-morbidities, and associated injuries.

Classification of the acetabular fractures according to Letournel classification was done by two surgeons (5-years’and 10-years’ experience). Variants, subtypes, and atypical types of acetabular fractures as described by Letournel was categorized under the corresponding types of the Letournel classification (five elementary types and five associated types). Failure to categorize the fracture into any of the types or subtypes of Letournel classification was considered to be unclassified fracture. Any discrepancy in the classification between the two surgeons was resolved by a joint discussion and by adjudication by the senior author (> 20-years’ experience in acetabular fracture surgery).

### Statistical analysis

Descriptive statistics: Means, standard deviations, medians, ranges, frequency, and percentages were calculated. Cohen’s kappa coefficients were used to measure interobserver reliability in determining fracture classification. Reliability is rated as ‘moderate’ for values between 0.41 and 0.60, as ‘substantial’ for values between 0.61 and 0.8 and as ‘excellent’ for values above 0.80.

## Results

In 2 consecutive years, 236 acetabular fractures in 235 patients were included in our study and no fracture was excluded. Table 1 demonstrates the basic demographics of the studied sample. In 214 (90.7%) fractures, we could assign a category according to Letournel Classification (53.7% elementary and 37% associated). Table 1 demonstrates the incidence of each fracture type in our series. Twenty-two fractures (9.3%) did not fit into any of the fracture types or subtypes according to Letournel classification. Table 2 described the unclassified fracture patterns. The interobserver reliability was 0.78.

**Table 1:** Basic demographics of the studied sample

Variable	*N* = 236
*Age/years*	
Mean ± SD	35.64 ± 13.9
*Sex*	
Male	197 (83.5%)
Female	39 (16.5%)
*Mechanism of injury*	
Road traffic accident (RTA)	194 (82.2%)
Fall from height	26 (9.8%)
Fall on ground	11 (4.6%)
Heavy object trauma	5 (2.1%)
Associated co-morbidity	26 (11%)
Isolated injury	145 (61.5%)
Associated musculoskeletal injury	78 (33%)
Associated non-musculoskeletal injury	13 (5.5%)

**Table 2:** The incidence of each fracture type in our series compared to other studies

Fracture pattern	Our study %	Letournel [36] %	Matta [37] %	Giannudis [38] %	Hutt [19] %
Anterior wall	1.3	1.6	1.2	1.7	0
Anterior with posterior hemitransverse	2.1	8.8	5.9	5	9
Posterior column with posterior wall	6.4	3.5	3.9	5.7	3
Posterior column	6.4	2.3	3.1	3.5	3
*T*	8.1	5.3	12.2	9.3	6
Transverse with posterior wall	8.5	20.6	23.5	17.4	4
Anterior column	9.3	3.9	4.7	3.9	6
Both column	11.9	27.9	33.3	21.7	22
Transverse	14.4	3.7	3.5	8.3	3
Posterior wall	22.4	22.6	8.6	23.6	9
Unclassified	9.3	NR	NR	NR	35

NR, Not reported

**Fig. 1:**
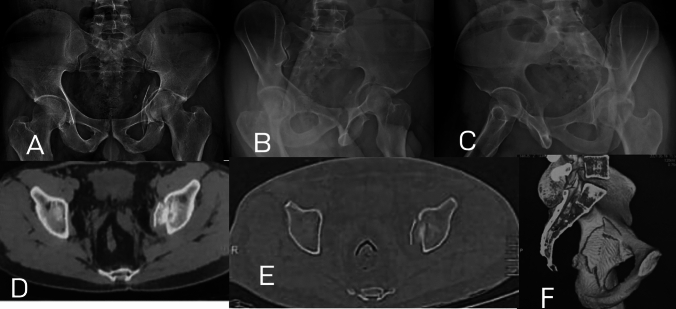
Pure quadrilateral plate fracture; **A** AP view, **B** Iliac Oblique view showing intact posterior column, **C** Obturator Oblique view showing intact anterior column with elevated plate of the quadrilateral surface, **D**, **E**;Axial CT cuts and **F;** 3D reconstruction showing displacement of the quadrilateral surface with intact both columns

In one case (4.5%), pure quadrilateral plate (Fig. 1), there was a complete separation of the quadrilateral plate from the anterior and posterior column. Bothe columns and walls were free. Elnahal et al. [20] defined quadrilateral plate fracture as complete or partial separation of the quadrilateral plate. It usually accompanies other fracture types, but in this case, both columns and wall were intact.

**Fig. 2:**
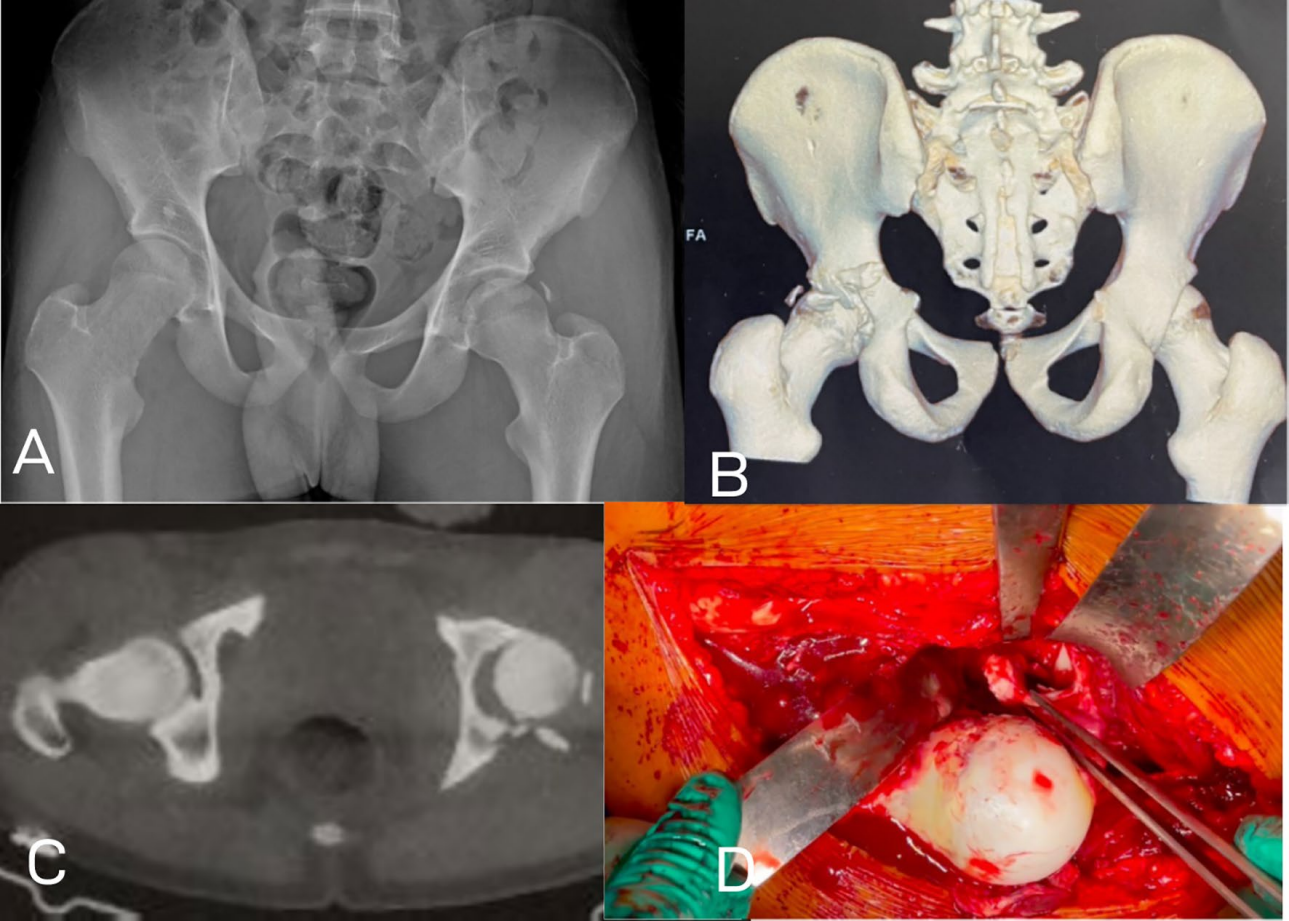
Labral avulsion; **A** AP radiograph showing sublaxed left hip with tiny posterosuperior bony fragment. **B** 3D reconstruction and **C** axial CT cut showing multipl tiny fragments of the posterior wall **D** intraoperative photo during surgical hip dislocation showing the lesion to be avulsion the posterior labrum with small bony rim attached

In one case (4.5%), there was a posterior fracture dislocation (Fig. 2). Preoperative CT showed sublaxed hip with very small posterior rim fragments and intraarticular fragments. Intraoperatively during surgical hip dislocation, the principal lesion was labral avulsion with small, tiny fragments of the posterior rim (Table 3).

**Table 3:** The unclassified fracture patterns

Pattern of unclassified fractures	*n* = 22
Pure quadrilateral plate	1 (4.5%)
Labral avulsion with tiny posterior wall rim	1 (4.5%)
Pure impaction injury with incomplete fracture lines	1 (4.5%)
Both column and post wall	1 (4.5%)
Anterior column and quadrilateral plate	4 (18.2%)
T with posterior wall	14 (63.8%)

**Fig. 3:**
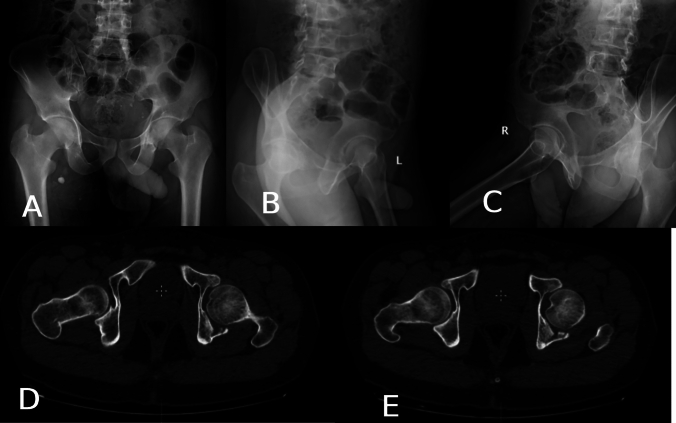
Pure impaction injury; **A** AP view, **B** Iliac oblique view, and **C** Obturator oblique view showing no detectable fracture lines in the left hip, **D**, and **E**, axial CT cuts showing large, impacted area of the articular surface of the posterior column with incomplete fracture lines not reaching the surface

In one case (4.5%), pure impaction injury (Fig. 3), there was only fractured and impacted articular surface with incomplete fracture lines not reaching the surface. Both columns and walls were intact.

**Fig. 4:**
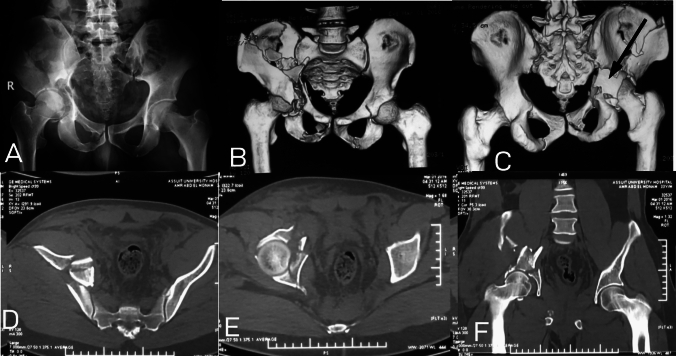
Both columns with posterior wall; **A–F** AP view and CT scan showing both columns fracture associated with posterior wall (arrow). The posterior wall in this case is the extended variant as described by Letournel

**Fig. 5:**
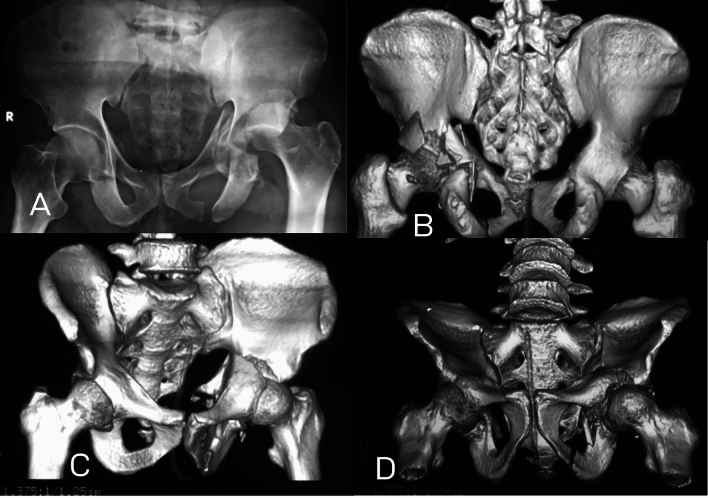
*T* with posterior wall

**Fig. 6:**
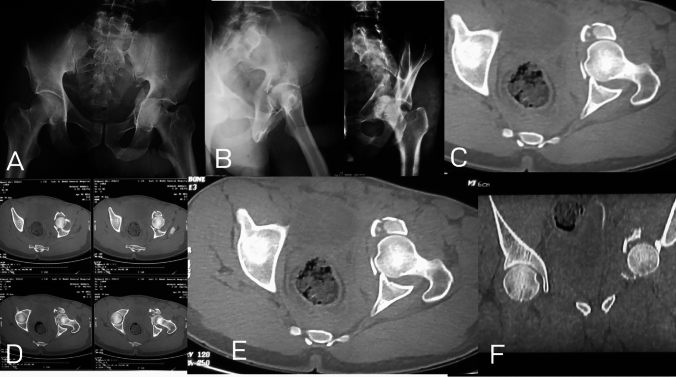
Anterior column with quadrilateral plate

In one case (4.5%), there was associated posterior wall fracture with both column fracture (Fig. 4). The posterior wall type in this fracture was atypical type as described by Letournel, extended posterior wall fracture. Similarly, we found association between posterior wall and *T* Fracture in 14 cases (63.8%) (Fig. 5). In four cases (18.2%), there were association between anterior column and quadrilateral plate (Fig. 6).

## Discussion

Letournel classification is considered the corner stone for classifying acetabular fractures. In his book, Letournel [21] extensively described each type of acetabular fractures. Transitional types between all types of acetabular fractures do exist.

Letournel classification was developed based on specific radiological landmarks on anteroposterior pelvic radiographs and Judeh (Obturator and iliac oblique) views.

Using 2D and then 3D computed tomography (CT) improved the reliability of Letournel classification and enabled better delineation of fracture lines and comminution zones within the acetabulum [22–25]. Recently, fracture mapping techniques based on 3D CT have  been widely used to elucidate fracture patterns in acetabular fractures [26, 27]. Moreover, The use of 3D printing models [28] and virtual reality (VR) [29] added to better understanding of the complex 3D anatomy of acetabular fractures.

Given the ongoing advances in fracture imaging techniques and the fact that Letournel classification might not be perfectly inclusive [30], there is increasing number of studies that reported “undescribed” or “unclassified fractures”. Boudissa et al. [31] and Ochs et al. [17] reported only 1% of acetabular fractures in their series to be unclassified. The percentage of unclassified fractures increased to 9% in the series of Mauffrey et al. [18] and to 20% by Herman et al. [32]. Hutt et al. [19] reported 35 fractures out of 100 (35%) to be unclassified by more than one researcher. Most of these fractures were anterior column fracture with associated quadrilateral plate involvement (65%). Herman et al. [32], in their cohort, reported 46 acetabular fractures out of 229 (20.1%) to be unclassified by Letournel classification as follows; anterior column with quadrilateral plate 18 (39.1%), posterior column with posterior wall 11 (23.9%), anterior column posterior hemitransverse with posterior wall 8 (17.4%), T with posterior wall 8 (17.4%), and anterior column with anterior wall 1 (2.2%). To overcome these drawbacks, Hermann et al. proposed a new classification for acetabular fractures;” there is no column” classification.

In this series, we reported 22 (9.3%) out of 236 cases of unclassified acetabular fractures. In concordance with Ovre et al. [33] and Hermann et al. [32], we reported association between posterior wall fracture with both columns in one case (4.5%) and with *T* fracture in 14 cases (63.8%). Ovre et al. [33] described a transitional type of fracture between transverse and *T*-fracture, inverse *T*-fracture. They reported the association of posterior wall fragment in 2 out of 6 inverse T fracture. Similarly, Hermann et al. [32], reported association between posterior wall fracture with *T*-, both columns and anterior column with posterior hemitransverse. Although, Letournel [21] in his book reported that 15.5% of both column fractures were associated with posterior wall fracture, he did not assign a subtype for this combination. On the other hand, he did not report the association between *T* fracture and posterior wall.

This association is important as it may alter the surgical approach in *T* fracture (anterior vs. posterior) or cause the addition of another approach (posterior) to the anterior approach in both-column fracture.

We reported 4 cases (18.2%) of anterior column with quadrilateral plate. Similarly, Hut et al. [19] and Hermann et al. [32], reported this type which represented (65%) and (39.1%) of their series of unclassified fractures respectively. With increasing frequency of acetabular fractures in the elderly and increasing the prevenance of osteoporosis, theses fractures are becoming more common nowadays [34]

Pure quadrilateral plate injury with intact both columns and walls was reported before as a case report of undescribed pattern of injury [9–11] In our case (4.5%), we followed the definition of Elnahal et al. [20] for quadrilateral plate fracture to be incomplete or complete separation of the quadrilateral plate from either the anterior or posterior columns or both. In our case, there was a complete separation from both the anterior and posterior column with displacement of the quadrilateral plate. Both columns and walls were intact.

Pure impaction of the articular surface of acetabulum is a rare injury that mandates special approaches as surgical hip dislocation [35] to access the impacted cartilage. Pascarella et al. [16] described a similar injury for articular impaction with incomplete fracture lines.

Letournel [21] described posterior wall fracture to range from few millimeters fracture of the rim to large posterior wall fragments. In our case, the tiny, small fragments were attached to the acetabular labrum which was detached from 1 to 5 o’clock of the left hip. We considered it bony avulsion of the labrum rather than a posterior wall fracture. Accordingly, it required different management through surgical hip dislocation and attachment of the avulsed labrum using suture anchors plus small spring plates for the small posterior wall fragments.

The value of defining a special category for the unclassified fractures is to draw the attention to unique pattern of fractures so it will not be overlooked, unique combinations which may require modification of the surgical approach or fixation technique or add a new one. Despite the increasing reports of unclassified fracture, Letournel classification is still the gold stone for classifying acetabular fractures [30]. Adding radiographic and CT-based modifiers as quadrilateral plate, articular impaction to Letournel classification can solve this issue of incomprehensiveness. Moreover, posterior wall can also be added as a radiological modifier to report the association of posterior wall involvement to fracture types other than posterior wall fracture type.

This study has several limitations. First, is the relatively small number of cases. However, we included all cases of acetabular fractures presented at our level-1 trauma center. Second, fractures were considered unclassified based only on Letournel classification. Some of the unclassified fractures in this study may be classifiable by other classification system. However, Letournel classification is the most popular and the most used classification system. Third, some of the fracture pattern we included in the unclassified type are not uncommon as *T* with posterior wall. However, being unclassified does not necessarily implies rarity. In concordance with this study, Hermann et al. [32] considered T with posterior wall acetabular fractures in their cohort to be unclassified by Letournel classification.

## Conclusion

Although Letournel classification was considered highly inclusive, a lot of studies reported the presence of unclassified or undescribed fracture patterns. These unique patterns may require special approaches or special fixation methods. However, this is not a call for a new classification for acetabular classification to include these new types. Subclassification or adding modifiers to Letournel classification can do the job.
